# Effective Treatment of Livedoid Vasculopathy With Oral Tofacitinib

**DOI:** 10.1002/ccr3.70536

**Published:** 2025-05-28

**Authors:** Parvin Mansouri, Nikoo Mozafari

**Affiliations:** ^1^ Medical Laser Research Centers, Academic Center of Education, Culture and Research Tehran University of Medical Sciences Tehran Iran; ^2^ Skin Research Center Shahid Beheshti University of Medical Sciences Tehran Iran; ^3^ Department of Dermatology Loghman Hakim Hospital, Shahid Beheshti University of Medical Sciences Tehran Iran

**Keywords:** baricitinib, JAK inhibitors, livedoid vasculopathy, tofacitinib

## Abstract

We present a woman with severe refractory livedoid vasculopathy who was successfully treated with tofacitinib (5 mg twice a day). The ulcers gradually healed over a period of 2–3 months without experiencing any adverse events over a 1‐year follow‐up period. Tofacitinib may be considered a safe and effective therapy for patients with livedoid vasculopathy.

## 1 Introduction

Livedoid vasculopathy is a chronic dermatological condition characterized by the recurrent appearance of erythematous purpuric papules and painful ulcers, primarily localized on the lower extremities, particularly the dorsum of the feet and ankles, which significantly impair quality of life [1, 2]. Livedoid vasculopathy is a rare thrombo‐occlusive vasculopathy characterized histologically by intraluminal fibrin deposition and thrombosis within the superficial dermal blood vessels. It exhibits a predilection for females, being three times more prevalent in females than in males [1]. Although the exact pathogenesis of livedoid vasculopathy (LV) remains elusive, it is hypothesized that increased local or systemic thrombotic activity, or decreased fibrinolytic activity, precipitates thrombus formation within small and medium‐sized dermal vessels [1].

The treatment of livedoid vasculopathy poses a significant challenge. While various reports have indicated favorable responses to treatments such as antiplatelet, anticoagulant, and fibrinolytic agents [2, 3], no single therapeutic approach proves universally effective for all patients. Therefore, there is a need for the development of novel therapeutic strategies. More recently, JAK inhibitor agents such as baricitinib or tofacitinib have demonstrated promising results in the treatment of refractory LV [4, 5].

In this study, we present a woman with severe refractory livedoid vasculopathy who was successfully treated with tofacitinib.

## 2 Case History/Examination

**FIGURE 1:**
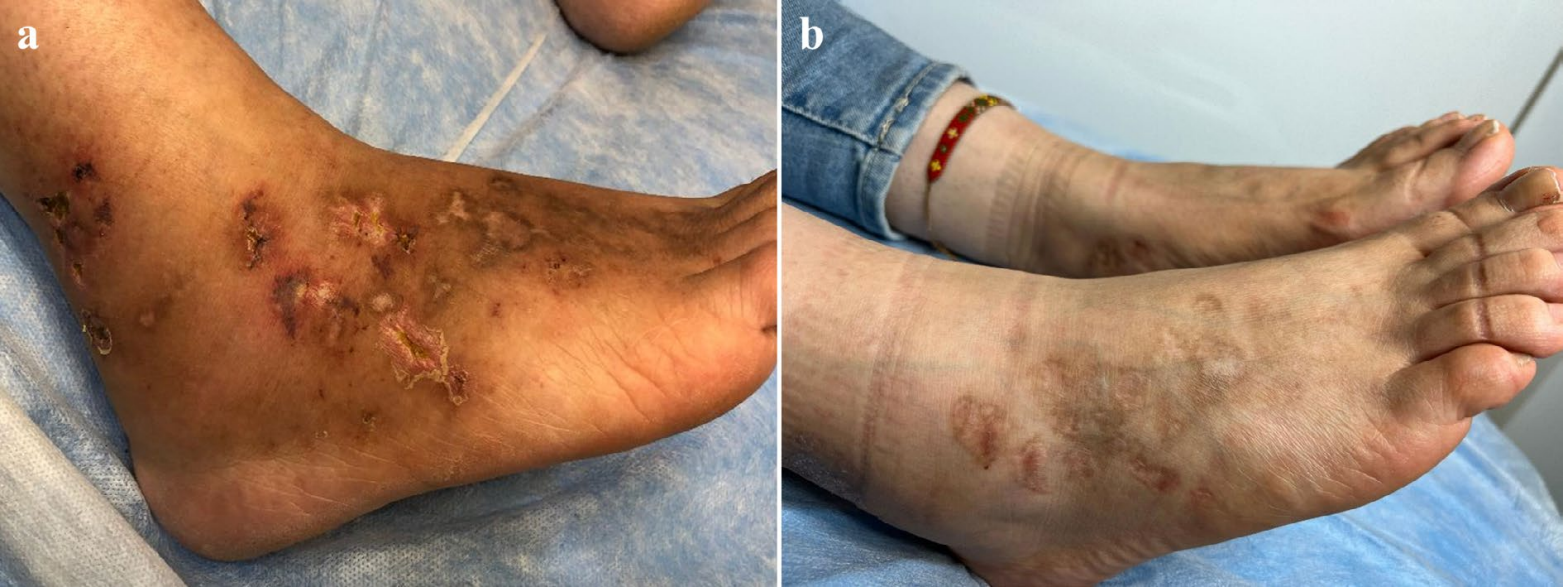
(a) Purpuric and punched‐out ulcerating lesions accompanied by atrophic scarring, telangiectasia, hemosiderin deposits, and hyperpigmentation over dorsum of feet and medial and lateral malleolus. (b) Two months after treatment, lesions completely healed with scarring.

A 32‐year‐old female patient presented with a 5‐year history of reticular discoloration and non‐healing ulcers on both legs (Figure 1a). She had been partially managed with hydroxychloroquine, pentoxifylline, and aspirin. However, 1 month prior to presentation, she experienced a flare‐up that did not respond to her previous medications. Clinical examination revealed painful, focal, purpuric ulcerating lesions accompanied by atrophic scarring, telangiectasia, hemosiderin deposits, and hyperpigmentation. Additionally, multiple painful, punched‐out ulcers surrounded by purpuric erythema were observed on the lateral malleoli and lower shins.

## 3 Methods (Investigations and Treatment)

A skin biopsy taken from a leg ulcer confirmed findings consistent with livedoid vasculopathy, including atrophic epidermis, fibrin deposition in the vessel walls, and hyaline thrombi in the vascular lumen of medium‐sized blood vessels. Laboratory parameters including coagulation factors, phospholipid antibodies, lupus anticoagulant, anticardiolipin antibodies, antinuclear antibodies (ANA), antineutrophil cytoplasmic antibodies (ANCA), cryoglobulins, cold agglutinins, protein C level, and factor V Leiden were within normal ranges or tested negative.

Prednisolone at a dosage of 30 mg per day was added to the patient's previous medications. The ulcers showed improvement upon initiation of prednisolone therapy, with several ulcers healing and leaving behind white atrophic stellate scars. However, ulcer recurrence was observed upon tapering the glucocorticoid dose to 10 mg per day. Subsequently, prednisolone was increased to 20 mg per day, and tofacitinib(Rhofanib by. Nanoalvand Corp, Iran) was administered at a dose of 5 mg twice per day, with discontinuation of other drugs.

## 4 Conclusion and Results (Outcome and Follow‐Up)

The ulcers completely healed after 2 months of this combined therapy (Figure 1b). Prednisolone was gradually tapered and discontinued over a period of 6 months, while tofacitinib was continued with a dose of 5 mg twice a day for 6 months; then it was tapered to a maintenance dose of 5 mg per day. The patient has been maintained on tofacitinib during follow‐up for 12 months without experiencing any relapse.

## 5 Discussion

The underlying mechanism of action of tofacitinib in refractory livedoid vasculopathy remains unclear [5]. Initially, livedoid vasculopathy was considered to be a vasculitis. However, the consistent failure to identify neutrophil infiltration in the blood vessels and the presence of fibrinoid necrosis led many authors to dispute this etiology [1, 6]. Therefore, livedoid vasculopathy is currently considered to be a coagulation disorder, distinctly separate from inflammatory vasculitis. The primary pathogenesis of livedoid vasculopathy is believed to involve hypercoagulability, encompassing thrombotic and microcirculatory phenomena. Pentoxifylline, aspirin, dipyridamole, and rivaroxaban are believed to enhance blood flow in capillaries and have been reported to demonstrate efficacy in clinical settings [2, 3].

However, some patients with livedoid vasculopathy do not respond to antithrombotic monotherapy, and aggressive immunosuppressive therapy may be necessary to manage disease activity. This observation suggests the possible involvement of an inflammatory response in the pathogenesis of livedoid vasculopathy [6]. Reports documenting successful treatment outcomes in livedoid vasculopathy with medications possessing anti‐inflammatory properties, such as anti‐TNF alpha inhibitors [7] and rituximab [8] further support the hypothesis implicating an inflammatory component in the pathogenesis of the condition.

The rationale for the use of tofacitinib in livedoid vasculopathy is based on its effects on two key pathogenic mechanisms: inflammation and the thrombotic pathways, which are explained as follows. Tofacitinib is a Janus kinase inhibitor approved for the treatment of inflammatory conditions such as rheumatoid arthritis and inflammatory bowel diseases. By inhibiting signal transducers and activators of transcription pathways involved in cytokine signaling, tofacitinib suppresses vascular inflammation by interfering with inflammatory cytokine signaling [9].

In myeloproliferative neoplasms with JAK2 mutations, such as essential thrombocythemia and myelofibrosis, the risk of thrombosis increases up to sixfold. This suggests that dysregulation of the JAK–STAT signaling pathway contributes to the overexpression of pro‐thrombotic markers, including von Willebrand factor and P‐selectin, leading to heightened vascular activation and an increased risk of thrombosis [10]. Notably, JAK–STAT inhibitors, such as ruxolitinib, have been shown to mitigate the upregulation of key pro‐thrombotic pathways and reduce leukocyte–endothelial adhesion [10].

In the context of livedo vasculopathy (LV), inflammation plays a significant role in its pathogenesis. Additionally, the upregulation of pro‐thrombotic pathways, as evidenced by elevated P‐selectin levels, has been observed in patients with LV [11]. This supports the hypothesis that JAK inhibitors may offer therapeutic benefits by downregulating these pathways and reducing endothelial activation, thereby alleviating symptoms in affected individuals.

The effectiveness of JAK inhibitors in the treatment of livedoid vasculopathy has been repeatedly reported in the literature [12]. A total of 17 patients diagnosed with ulcerating livedoid vasculopathy were reported to have been treated with JAK inhibitors. Fourteen patients received baricitinib [13, 14, 15, 16], two patients received tofacitinib [5, 17] and one patient was treated with abrocitinib [18] (Table 1).

**TABLE 1:** Clinical data of reported cases of livedoid vasculopathy treated with JAK inhibitors.

Author	Number of reported patient	Age (years)	Sex	Prior treatment	Current treatment	Outcome	Time to reach remission	Duration of treatment (months)	Adverse effects
Han and Tu [4]	8	8–36	3 M/5 F	Aspirin, GC, thalidomide, rivaroxaban, enoxaparin, Chinese traditional anti‐inflammatory drugs	Baricitinib 2 mg daily	Significant improvement in erythema, ulceration, and pain	3–13 weeks	2.5–7	None
Song and Tu [15]	3	8/26/26	M/M/F	GC, thalidomide, rivaroxaban, and aspirin	Baricitinib 2 mg daily	Complete healing	1–6 months	4–18	None
Zhang et al. [13]	1	14	F	Chinese medicine anticoagulants and GC	Baricitinib 4 mg daily	Significant improvement of ulcers	7 weeks	3	None
Peñuelas et al. [16]	1	48	F	Nifedipine, aspirin, GC, and etanercept	Baricitinib 4 mg daily	Healing of ulcers	4 weeks	4	None
Xiao et al. [14]	1	26	F	GC, thalidomide, and aspirin	Baricitinib 4 mg	Significant improvement of ulcers	12 weeks	10.5	None
Jia et al. [5]	1	17	M	Colchicine, thalidomide, dipyridamole, rivaroxaban, aspirin, and GC	Tofacitinib 5 mg twice a day	Complete healing	4 weeks	13	None
Rudrakar and Kumar [17]	1	43	F	Antiplatelets, GC, MTX, and warfarin	Tofacitinib 5 mg twice a day	Improvemet in pain and ulceration	2 months	12	None
Chen et al. [18]	1	31	F	NR	Abrocitinib 100 mg daily	Complete remission	6 weeks	3	None

Abbreviations: GC, glucocorticoids; NR, not reported.

In the largest case series, Han and Tu [4] conducted a retrospective study to assess the clinical efficacy of baricitinib in the treatment of eight patients with livedoid vasculopathy resistant to conventional therapies. The study cohort comprised five females and three males, aged 8–36 years. Patients were treated with a daily dose of 2 mg of baricitinib. All patients demonstrated clinical improvements within 3–13 weeks, with significant improvement observed in erythema, ulceration, and pain [4].

In the remaining reported cases, patients with livedoid vasculopathy refractory to conventional treatments such as antiplatelet therapy, anticoagulants, or etanercept were found to respond to baricitinib at doses of 2–4 mg orally daily, and significant improvements were observed after 4–12 weeks [13, 14, 15, 16]. In these reported cases, baricitinib treatment was administered as the sole medication, following cessation of other drugs. The authors demonstrated that baricitinib monotherapy exhibited good efficacy without necessitating additional treatments. Furthermore, baricitinib treatment was well‐tolerated, and no side effects related to baricitinib, such as upper respiratory infections, herpes simplex virus infection, folliculitis, tuberculosis, malignant tumors, or deep vein thrombosis, were observed in this cohort of patients.

The therapeutic efficacy of tofacitinib in the management of livedoid vasculopathy has been documented in only two case reports [5, 17]. Jia et al. and Rudrakar and Kumar independently described cases of patients with livedoid vasculopathy refractory to antiplatelet agents and anticoagulants, who achieved complete resolution of their symptoms after several months of treatment with tofacitinib at a dosage of 5 mg orally twice daily [5, 17]. Similar to the reported case, our patient was successfully treated with tofacitinib without experiencing any adverse events over a 1‐year follow‐up period.

In conclusion, our case demonstrates that tofacitinib may be considered a safe and effective therapy in patients with livedoid vasculopathy. However, as the use of tofacitinib is currently limited to case series, further studies are warranted to confirm its efficacy and safety in this patient population.

## Author Contributions

**Parvin Mansouri:** conceptualization, data curation. **Nikoo Mozafari:** conceptualization, data curation, writing – original draft, writing – review and editing.

## Disclosure

We declare that none of the authors listed on the manuscript are employed by a government agency that has a primary function other than research and/or education. None of the authors submitting this manuscript are acting as an official representative or on behalf of the government.

## Consent

The patient in this manuscript gave written informed consent for the publication of her case details.

## Conflicts of Interest

The authors declare no conflicts of interest.

## Data Availability

The data presented in this study are available on request from the corresponding author.
